# Nutritional Status, Dietary Intake, and Relevant Knowledge of Adolescent Girls in Rural Bangladesh

**DOI:** 10.3329/jhpn.v28i1.4527

**Published:** 2010-02

**Authors:** Nurul Alam, Swapan Kumar Roy, Tahmeed Ahmed, A.M. Shamsir Ahmed

**Affiliations:** ^1^ Public Health Sciences Division; ^2^ Clinical Sciences Division; ^3^ Nutrition Programme, Clinical Sciences Division, ICDDR,B, GPO Box 128, Dhaka 1000, Bangladesh

**Keywords:** Adolescents, Dietary knowledge, Food frequency, Girls, Nutrition, Bangladesh

## Abstract

This study estimated the levels and differentials in nutritional status and dietary intake and relevant knowledge of adolescent girls in rural Bangladesh using data from the Baseline Survey 2004 of the National Nutrition Programme. A stratified two-stage random cluster-sampling was used for selecting 4,993 unmarried adolescent girls aged 13–18 years in 708 rural clusters. Female interviewers visited girls at home to record their education, occupation, dietary knowledge, seven-day food-frequency, intake of iron and folic acid, morbidity, weight, and height. They inquired mothers about age of their daughters and possessions of durable assets to divide households into asset quintiles. Results revealed that 26% of the girls were thin, with body mass index (BMI)-for-age <15^th^ percentile), 0.3% obese (BMI-for-age >95^th^ percentile), and 32% stunted (height-for-age ≤2SD). Risks of being thin and stunted were higher if girls had general morbidity in the last fortnight and foul-smelling vaginal discharge than their peers. Consumptions of non-staple good-quality food items in the last week were less frequent and correlated well positively with the household asset quintile. Girls of the highest asset quintile ate fish/meat 2.1 (55%) days more and egg/milk two (91%) days more than the girls in the lowest asset quintile. The overall dietary knowledge was low. More than half could not name the main food sources of energy and protein, and 36% were not aware of the importance of taking extra nutrients during adolescence for growth spurt. The use of iron supplement was 21% in nutrition-intervention areas compared to 8% in non-intervention areas. Factors associated with the increased use of iron supplements were related to awareness of the girls about extra nutrients and their access to mass media and education. Community-based adolescent-friendly health and nutrition education and services and economic development may improve the overall health and nutritional knowledge and status of adolescents.

## INTRODUCTION

Childhood undernutrition, highly prevalent in South Asia (1), continues to persist throughout adolescence but little attention has been given to undernutrition of adolescents perhaps for the belief that adolescents are a low-risk group. Stunting in adolescence is 32% in India, 36% in Bangladesh, and 47% in Nepal, and low body mass index (BMI) is 53% in India, 50% in Bangladesh, and 36% in Nepal (2). In Bangladesh, 25–27% of adolescent girls are anaemic (haemoglobin <12 g/dL) (3, 4), and 30% in the age-group of 14–18 years are iron-deficient (serum transferrin saturation [TS] <15%) (5, 6). Half (47–54%) of schoolgoing children are vitamin A-deficient (5, 6). The level of zinc deficiency in adolescence is unknown. The average per-capita energy intake by rural adolescent girls is 81% of the recommended dietary allowance (RDA) for age (7, 8). Protein, iron, and calcium are important for growth spurt and skeletal development in adolescence. More than 60% of schoolgirls aged 10–16 years in Dhaka city consume protein, iron, and calcium less than 75% of the RDA for age (6). The high prevalence of chronic energy and micronutrient deficiencies of today's adolescent girls is directly linked to the quality of the next generation. Without addressing these deficiencies, the vicious cycle of inter-generational undernutrition, chronic diseases, and poverty perpetuates (9, 10).

Adolescence is a unique intervention point in the life-cycle for a number of reasons (11). Early adolescence after the first year of life is the second critical period of rapid physical growth and changes in body composition, physiology, and endocrine. Rapid growth and changes heighten their nutritional requirements and risks of undernutrition. Parents simply need to provide more nutrients and emotional support. Adolescence offers the last opportunity to intervene and recover growth faltered in childhood and also support growth spurt and skeletal development to break the vicious cycle of inter-generational undernutrition (9, 10).

In developing countries, factors associated with undernutrition of adolescents are: poor household economic condition, periodic food-shortage, child-labour (marker of household income-poverty), burden of disease, poor knowledge about long-term consequences of undernutrition of adolescents, quantity and quality of food, and access to health and nutrition services (12). In Bangladesh, low family income, education, and periodic food-shortage were associated with inadequate dietary intake (7), which might have led to undernutrition. Previous studies have either ignored the burden of disease or estimated the burden of disease ignoring reproductive morbidity of unmarried adolescent girls (4, 5). The present study estimates the burden of disease, consisting of general and reproductive morbidity and its nutritional consequences, in addition to other factors.

Dietary knowledge and access to resources are critical to improve health and nutrition in a sustainable way. Adolescence is the time to learn and adopt healthy habits to avoid many health and nutritional problems later in life (12). Adolescents have more easy access to health and nutrition information through schools, recreational activities, and mass media than they have later in their lives (11). Particularly, health and nutrition knowledge and healthy habits of female adolescents will have critical roles to play in maintaining future family health and nutrition. No study has so far ever assessed dietary knowledge of adolescents, which this study has done for a number of factors.

To combat the high prevalence of micronutrient deficiencies, the Government of Bangladesh recommends supplementation of micronutrients (5, 13). The National Nutrition Programme of the Bangladesh Government, through area-based Adolescent Girls Forum, provides health and nutrition education and iron supplements to unmarried adolescent girls (3). The coverage of iron supplement in the programme and non-programme areas and its overall determinants are little known. This study also estimated the coverage for a number of factors to inform nutrition promoters to plan action.

Nutrition promoters need information on levels and differentials in undernutrition of adolescents, burden of disease, dietary knowledge, and intake and coverage of iron supplement to meet the needs of those who need them most. The objectives of this study were to provide them with such information that would assist in planning for remedial actions to address health and nutrition needs of adolescent girls in Bangladesh.

## MATERIALS AND METHODS

This study used data from the Baseline Survey 2004 of the National Nutrition Programme (NNP) conducted in rural areas of Bangladesh. The NNP is the successor to the Bangladesh Integrated Nutrition Project (BINP), which was launched in 1994. The BINP expanded its activities in phases and continued up to 2003. The community-based nutrition centres run by female community nutrition promoters (CNPs) were the focus of the BINP activities. The CNPs imparted health and nutrition education to mothers of children aged less than two years (under-two children), adolescent girls, and pregnant women; monitored growth of under-two children; and provided food supplements to severely-underweight and growth-faltered under-two children and to undernourished pregnant women (BINP implementation plan 1994). They organized never-married adolescent (out-of-school and schoolgoing) girls of the community to form Adolescent Girls Forum and to hold its meeting monthly to discuss their health, nutrition and social safety issues and distributed iron and folic acid tablets to the participants of the meeting.

A stratified two-stage random cluster-sampling was used for selecting 5,028 never-married adolescent girls aged 13–18 years from 708 clusters in 113 upazilas (subdistricts) spread in all six administrative divisions (strata) of Bangladesh. The first stage was the random selection of clusters from cluster lists in programme and non-programme upazilas where each cluster contains about 300 households. The second stage was the random selection of nine unmarried adolescent girls from each cluster of the NNP and comparison upazilas and six unmarried adolescent girls from each cluster of the BINP upazilas for interview. Upazilas were grouped into BINP upazilas, NNP upazilas (where NNP activities were yet to start), and comparison upazilas (selected to assess the effects of NNP interventions later). Analysis included 4,993 never-married adolescent girls aged 13–18 years (excluded were 35 girls with flagged BMI-for-age or height-for-age z-score).

Female interviewers, trained by social scientists and nutritionists, visited adolescent girls at their homes with a structured questionnaire to obtain consent of parents and to collect data on health and nutrition status of the adolescent girls. Field supervisors checked work of the field interviewers daily to ensure the quality of data collected in the field. Interviewers inquired girls about their education, occupation, and access to electronic and print media. Education was measured with grade passed and categorized as: up to primary (passed grade 5 or less) and secondary or above (passed grade 6 or more). Access to media was considered high if she had listened to radio or watched television at least weekly or read a newspaper or magazine (no matter how frequent it was); otherwise, it was considered low.

To assess their dietary knowledge, a checklist of food items (rice/wheat, potato, pulses, meat, fish, milk, eggs, vegetables, and fruits) was used. Girls were asked to name the check-listed foods that provide mainly energy, protein, vitamin, and minerals. They were asked if they were aware of the importance of taking extra nutrients during adolescence and use of iron supplements to assess their dietary knowledge and intake. Despite the possibility of recall bias, the girls were asked about the seven-day food-frequency questions to assess eating patterns of energy, protein, fat, and vitamin- and mineral-rich food items. A checklist was used for recording general morbidity symptoms they had experienced in the preceding two weeks. The checklist included fever, cough/cold, diarrhoea/dysentery, stomachache, respiratory problems, ear and eye problems, skin problems, and others. The girls were asked privately about menstrual irregularities, avoidance of any food during last menstruation, and passage of white discharge with foul smell. Self-reported morbidity symptoms were taken at face value.

Weight, height, and mid-upper arm circumference (MUAC) of the girls were measured by Uniscales, locally-made height scales and TALC (Teaching Aid at Low Cost) tape respectively. Weight and height were used for computing BMI and height-for-age z-score using the WHO growth reference for school-age children and girls (14, 15). BMI-for-age below the 5^th^ percentile value of the WHO growth reference for girls refers to severely thin and BMI-for-age in between the 5^th^ and the 15^th^ percentile value refers to moderately thin. BMI-for-age below the 15^th^ percentile value and height-for-age z-score <-2SD were used in measuring the prevalence of thinness and stunting respectively.

Mothers were inquired about age or date of birth of their daughters and household possessions of durable items. A local calendar was used for determining age or date of birth accurately. Misreporting, particularly under-reporting of age, is likely and so are the underestimation of the prevalence of stunting and thinness. Household durables included bed-stand, chair, table, cupboard, radio, television, bike (including motor bike), land owned, wall material of the main dwelling unit (mud-floor accounted for 96% and tin-roof accounted for 94%, and these were, therefore, excluded from the analysis), and type of toilet used. The principal components analysis of household durable assets retained one factor and assigned factor score to each household (16). The higher the score the higher was the number of household assets, indicating better the long-term economic status of the household. The factor score was used for dividing the households into quintiles—from the lowest 20% to the highest 20%. The higher household asset quintile reflects higher economic status of the household.

### Analysis of data

Bivariate and logistic regression techniques were used for estimating the differentials in nutrition status, dietary consumption, and knowledge and intake of iron supplement. The differentials included age, education, morbidity, access to mass media, household asset quintile, and type of upazila (proxy for exposure to the community-based nutrition education project). Bivariate differentials are shown in terms of prevalence (in %) and mean of seven-day food intake. Logistic regression (Stata, version 8 with adjustment for clustering of nutrition status, intake by and knowledge of girls of the same cluster) was used in estimating the differentials, controlling simultaneously for others and expressed as odds ratios.

## RESULTS

### Characteristics of adolescent girls

The sampled girls were mostly young because some older ones got married and did not fit the inclusion criteria (Table 1). A very few (4%) have never attended schools, and 68% had education up to grade 6 or above. Two-thirds (66%) were students, and the remaining were doing household chores (30%) or economic activities (4%). Two-fifths (41%) did not have regular access to the mass media.

**Table 1.** Levels and differentials in prevalence (%) and odds ratio of severe and moderate thinness among never-married adolescent girls aged 13–18 years (n=4,993) for different characteristics

Characteristics	% of adolescent girls	Thinness (BMI <15^th^ percentile)		Stunting (HAZ <-2SD)	
		Prevalence (%)	Adjusted OR†	Prevalence (%)	Adjusted OR†
Age (years)					
13	20.2	30.7	1.00	15.9	1.00
14	23.7	23.0	0.73** (0.59–0.90)	26.4	2.18** (1.68–2.81)
15	21.8	19.6	0.63** (0.49–0.80)	34.2	3.48** (2.68–4.51)
16	15.9	23.4	0.77* (0.59–0.99)	42.7	5.61** (4.25–7.41)
17	10.2	28.4	1.05 (0.79–1.41)	43.9	5.74** (4.28–7.71)
18	8.1	33.0	1.27 (0.93–1.74)	48.3	6.67** (4.93–9.03)
Morbidity in the last two weeks					
No	66.5	24.5	1.00	31.0	1.00
Yes	33.5	27.0	1.17* (1.00–1.36)	34.2	1.03 (0.88–1.21)
Foul-smelling vaginal discharge					
No	73.9	22.4	1.00	31.4	1.00
Yes	20.7	26.7	1.21* (1.02–1.44)	33.5	1.20* (1.01–1.44)
No menarche yet	5.5	58.0	5.40** (3.97–7.36)	37.3	2.86** (1.98–4.15)
Knew need of extra nutrients					
No	35.7	25.0	1.00	32.8	1.00
Yes	64.3	25.4	1.01 (0.85–1.20)	31.7	0.91 (0.78–1.06)
Exposure to mass media					
Low	41.2	25.3	1.00	34.7	1.00
High	58.8	25.3	0.99 (0.83–1.17)	30.3	0.85 (0.73–1.00)
Education level					
Up to primary	31.6	25.6	1.00	37.2	1.00
Secondary +	68.4	25.1	1.05 (0.86–1.27)	29.8	0.60** (0.51–0.72)
Household asset quintile					
Lowest	20.1	24.9	1.00	34.5	1.00
Second lowest	20.2	25.0	0.87 (0.68–1.11)	33.6	1.09 (0.88–1.35)
Middle	20.2	24.3	0.93 (0.72–1.19)	31.9	0.99 (0.78–1.25)
Fourth	19.9	25.7	0.98 (0.76–1.27)	29.0	0.85 (0.66–1.09)
Highest	19.7	26.3	1.05 (0.80–1.38)	31.6	1.01 (0.78–1.31)
Survey upazila					
NNP	46.1	26.3	1.00	31.6	1.00
BINP	38.3	24.3	0.95 (0.80–1.11)	32.4	1.05 (0.91–1.21)
Comparison	15.5	24.7	0.90 (0.72–1.12)	33.2	1.06 (0.86–1.30)
All	100	25.3		32.2	
Modelχ^2^ (with df)		174.8 (17), p<0.001		317.9 (17), p<0.001

The dependent variable equals to 1 if BMI of a girl was below the 15^th^ percentile value of BMI-for-age or height-for-age <-2SD in WHO reference for girls, 0 otherwise;

†OR was adjusted for clustering of nutrition status of girls of the same clusters;

*p<0.05,

**p<0.01 (compared with reference category);

BINP=Bangladesh Integrated Nutrition Project;

BMI=Body mass index;

df=Degree of freedom;

HAZ=Height-for-age z-score;

NNP=National Nutrition Programme;

OR=Odds ratio;

SD=Standard deviation;

WHO=World Health Organization

### Perceived morbidity

The burden of disease measured with self-reported morbidity was quit high; 33% of the girls had one or more morbidity symptoms in the last two weeks (Table 2). Morbidity symptoms in order of prevalence were: fever (17%), followed by cough and cold (8%), stomachache (4%), diarrhoeal diseases (3%), and skin problems (1%). The burden of reproductive morbidity was unacceptably high; 10% had irregular menstruation, and 23% had foul-smelling vaginal discharge (an indicator of symptomatic reproductive tract infection [RTI]). In total, 276 (5%) adolescent girls, mostly younger in age, had not reached menarche before the survey date (pre-pubertal girls). Girls who had reported general morbidity in the preceding two weeks were more likely to have had RTI and vice versa than girls who had not reported (prevalence of co-morbidity).

**Table 2.** Self-reported general and reproductive morbidity in adolescent girls

General morbidity symptoms in the last two weeks								Foul-smelling vaginal discharge*
None	Any symptom	Fever	Cough/cold	Diarrhoea	Stomachache	Skin problem	Others	
66.5	33.5	16.9	8.0	2.6	3.8	1.4	13.4	22.9

*Among girls who reached menarche before the date of the survey

### Nutritional status

Nutritional status was measured with BMI-for-age of girls and height-for-age z-score. The prevalence of undernutition in the sampled adolescent girls was high; 9% were severely thin (below the 5^th^ percentile value of BMI-for-age for girls), 16% moderately thin (in between the 5^th^ and the 15^th^ percentile value of BMI-for-age), and 0.3% obese (above the 95^th^ percentile value) (Table 1). The age pattern of the prevalence of (severe and moderate) thinness followed the U-shaped curve. The prevalence of thinness was higher in early and late adolescence (it was 31% at the age of 13 years, fell to 20% at the age of 15 years, and increased to 33% at the age of 18 years). Of the pre-pubertal girls, 58% were thin, suggesting that undernutrition had delayed puberty. Exclusion of the pre-pubertal girls from the analysis did not change the results. The risk of being thin was significantly higher if girls had experienced general morbidity in the past two weeks or had experienced symptomatic RTI than if girls had not experienced either general morbidity or symptomatic RTI. Her knowledge about need for extra nutrients in adolescence, exposure to mass media, education, and household asset quintile were not associated with the risk of being thin.

Stunting (measured with height-for-age z-score <2SD) was prevalent among 32% of the girls, and its prevalence increased with increase in age. Girls with symptomatic RTI and lower education were more likely to be stunted than their counterparts. Knowledge of girls about the need for extra nutrients in adolescence, exposure to mass media, and household asset quintile were not associated with the prevalence of stunting.

### Seven-day food-frequency

Consumption of staple food (rice or wheat) in the last seven days was universal with no difference between the asset quintiles (data not shown). Consumptions of non-staple food items, such as meat, eggs, *dal* (lentils), fruits, and leafy vegetables, were not frequent in rural areas (Table 3). Half of them did not eat meat and milk each, and 40% did not eat eggs at all. The average days of consumption in a week were 3.4 for fish, 0.6 for meat, 1.1 for eggs, 1.9 for milk, 1.7 for *dal* and leafy vegetables each, and 2.3 for fruits. The availability and consumption of fruits is quite seasonal in rural Bangladesh.

**Table 3.** Weekly pattern of consumptions of selected food items by adolescent girls

Food item	Frequency of consumption, days per week				Mean days
	0 (%)	1–3 (%)	4–5 (%)	6–7 (%)	
Fish	9.1	34.1	15.7	41.1	3.4
Meat	50.5	45.0	3.2	1.3	0.6
Eggs	39.8	47.3	6.7	6.2	1.1
Milk	50.0	21.2	4.0	24.7	1.9
*Dal* (lentils)	25.3	52.6	9.3	12.7	1.7
Leafy vegetables	19.8	55.9	13.2	11.0	1.7
Fruits	24.0	41.3	11.4	23.3	2.3

To estimate the economic differentials in consumptions of protein- and fat-rich foods, we grouped together fish and meat (main sources of animal protein) and egg and milk (main sources of animal fat). The mean frequencies of eating fish/meat and egg/milk were 4.7 and 3.3 days respectively (Table 4). The household asset quintile correlated strongly with average days of consumptions; girls of the households in the highest asset quintile ate fish/meat 2.1 (55%) days more in one week and egg/milk two (91%) days more than girls of the households in the lowest asset quintile.

**Table 4.** Household asset quintile and mean days (and 95% confidence interval) of eating fish/meat and egg/milk by unmarried adolescent girls in the last seven days

Household asset quintile	Mean days of consumption		No. of adolescent girls
	Fish/meat Mean (95% CI)	Egg/milk Mean (95% CI)	
Overall	4.7 (4.7–4.8)	3.3 (3.2–3.4)	4,993
Lowest	3.8 (3.7–4.0)	2.2 (2.0–2.3)	1,003
Second	4.1 (4.0–4.3)	2.9 (2.7–3.1)	1,011
Middle	4.6 (4.5–4.8)	3.5 (3.3–3.7)	999
Fourth	5.2 (5.1–5.3)	3.8 (3.6–4.0)	994
Highest	5.9 (5.8–6.0)	4.2 (4.0–4.4)	986
Highest:lowest ratio	1.55	1.91	

CI=Confidence interval

### Dietary knowledge and intake of iron supplement

Knowledge of adolescents on common food sources of energy and protein was limited. When asked to name main food sources of energy, 31% mentioned rice and 12% mentioned wheat, and these are correct responses (data not shown). Less than half correctly named high-protein foods: *dal* (lentils) (21%), meat (32%), and fish (43%). Knowledge on vitamin- and mineral-rich foods was common; 75% mentioned vegetables, and 51% mentioned fruits as vitamin- and mineral-rich foods.

Two-thirds (64%) of the girls were aware of the need to take extra nutrients during adolescence to attain growth spurt (Table 5). Factors positively associated with the knowledge were their age, education, access to mass media, and the household asset quintile. This knowledge was more prevalent in the BINP upazilas than in the new NNP upazilas. The intake of iron supplements was higher (21%) in the BINP upazilas than in the new NNP upazilas (8%) and comparison upazilas (5%). However, the difference between the new NNP and the comparison upazilas was not statistically significant. The intake of iron increased with increase in age, education, knowledge about need to take extra nutrients, and access to mass media. The household asset quintile was not associated with the intake of iron.

**Table 5.** Percentage and odds ratio of knowing the importance of taking extra nutrients and taking iron supplements during adolescence for different characteristics

Characteristics	Unaware of extra nutrients		Intake of iron supplements	
	Crude %	Adjusted OR†	Crude %	Adjusted OR†
Age (years)				
13–14	55.3	1.00	8.6	1.00
15–16	68.2	1.41** (1.21–1.66)	13.7	1.55** (1.24–1.95)
17–18	77.9	2.13** (1.70–2.67)	19.1	2.14** (1.63–2.81)
Knew need of extra nutrients				
No		Not applicable	9.3	1.00
Yes			14.7	2.43** (1.90–3.12)
Exposure to mass media				
Low	52.7	1.00	6.3	1.00
High	72.4	1.63** (1.40–1.90)	15.9	1.24* (1.01–1.53
Education level				
Up to grade V	44.8	1.00	8.6	1.00
Grade VI or above	73.3	2.13** (1.80–2.54)	14.3	1.36* (1.03–1.79)
Household asset quintile				
Lowest	47.5	1.00	9.4	1.00
Second lowest	60.0	1.21 (0.99–1.48)	10.7	0.92 (0.66–1.30)
Middle	64.4	1.35** (1.07–1.70)	13.3	1.12 (0.80–1.56)
Fourth	69.5	1.45** (1.16–1.82)	14.3	1.13 (0.79–1.61)
Highest	80.5	2.01** (1.56–2.60)	14.6	0.83 (0.58–1.19)
Survey upazila				
NNP	62.5	1.00	8.4	1.00
BINP	66.6	1.34** (1.12–1.60)	20.6	3.53** (2.74–4.54)
Comparison	64.0	1.11 (0.88–1.38)	4.6	0.83 (0.51–1.36)
All	64.3		12.5	
Model χ^2^ (with df)		361.6 (10), p<0.001		244.6 (11), p<0.001

The dependent variable equals to 1 if a girl knew the need of taking extra nutrients during adolescence, took iron supplement, 0 otherwise;

†OR was adjusted for clustering of nutrition knowledge of girls of the same clusters;

*p<0.05,

**p<0.01 (compared with reference category);

BINP=Bangladesh Integrated Nutrition Project;

CI=Confidence interval;

df=Degree of freedom;

NNP=National Nutrition Programme;

OR=Odds ratio

## DISCUSSION

The results of the study revealed that, in rural Bangladesh, the prevalence of thinness and stunting among the adolescent girls aged 13–18 years was widespread and persistent. Severe thinness was 9%—lower than 16% found in schoolgirls aged 10–16 years in Dhaka city in 1995 (6). This difference is partly due to disproportionate distribution of age and age at onset of puberty of the girls in these two studies. Consistent with the findings of other studies (6, 17), the prevalence of thinness and stunting increased with increase in age. Other risk factors of thinness and stunting were self-reported general morbidity and symptomatic RTI, which were overlooked in previous studies. One may argue that self-reported morbidity was the effects rather than the causes of being thin or stunted. A foul-smelling vaginal discharge is usually generated from an infection, and its prevalence was unacceptably high among the unmarried adolescent girls (23%) which challenged our conviction that unmarried girls are at a lower risk of RTI. Further study is needed to verify self-reported foul-smelling vaginal discharge and find the possible causes. It might be associated with some harmful practices, such as use of old unclean clothes to manage menstruation blood, which is common among 42% of Bangladeshi adolescent girls (18). Nevertheless, effective preventive measures and treatment on time may lower the risk of being thin and stunted in the study population.

Although education was not associated with the risk of being thin, girls with higher education were less likely to be stunted than girls with lower education (odds ratio=0.60, 95% confidence interval [CI] 0.51–0.72). Household economic condition (measured by the asset quintile) was not associated with the risk of being thin or stunted. This could happen for a number of reasons. The seven-day food-frequency data showed no difference in the intake of rice/wheat (staple diet) among economic groups. Selectivity can be another possible reason; healthier girls looked mature and belonged to wealthier households and got married at a higher rate at earlier age than girls of poor households. Some like to look slim to improve body image and may have lowered the economic differential in thinness and stunting. Last but not the least is the frailty; girls of the poor households died off at a higher rate leaving behind the healthy ones and underestimating the economic differentials in the cross-sectional survey. Only a longitudinal study can resolve the frailty issue.

We explored the dietary patterns among rural girls in relation to household economic condition (measured with asset quintile). Food items, such as fish, meat, eggs, and milk, are the major sources of animal-protein, calcium, and vitamin A. Half of them could not have meat, eggs, or milk in a week. Girls of the households in the highest asset quintile did consume fish/meat 2.1 (55%) days more, and eggs/milk two (91%) days more than the girls of the households in the lowest asset quintile. The difference in intake of quality food between income-groups was reported for schoolgirls in Dhaka city (6). The large gap in intake of quality foods between the rich and the poor urges for interventions to reduce the gap (Millennium Development Goal 1). Both short-term (such as wider social safety-nets) and long-term (such as economic or livelihoods security) interventions are needed to minimize the rich-poor gap in consumptions of quality (animal protein and fat-rich) foods.

Iron deficiency is common among adolescent girls in Bangladesh (5, 6), as large-scale food fortification is not operating in the country. The Government of Bangladesh recommends supplementation of micronutrients to combat micronutrient deficiencies. Our results showed that, compared to the NNP upazilas, intake of iron supplements was significantly higher (21% opposed to 8%) in the BINP upazilas. This might be the effect of the distribution of iron and folic acid in the BINP community (19). The other factors associated with the increased intake of iron were knowledge on nutrition, education, and access of girls to mass media, suggesting that multi-sector approaches are needed to improve the coverage of iron supplement. Knowledge directly impacts health and nutrition, often for better sustainability. The findings of this study revealed that the majority (>57%) of the rural adolescent girls needed basic dietary knowledge, and 36% were not aware of the importance of nutrition in adolescence to support their growth spurt and skeletal development. Boys need the same dietary and nutrition knowledge, and they are almost twice as malnourished as girls (2).

Widespread undernutrition, micronutrient deficiency, low dietary knowledge, and low coverage of iron supplement among adolescents are concerns for public-health nutrition in Bangladesh. Adolescents are not the sole decision-makers. Parents, particularly mothers, often make decisions on their behalf, and they need to be sensitized about diet and nutritional needs in adolescence and adverse effects of undernutrition of adolescents to change their mindset. The CNPs, with appropriate communication materials, can do the job effectively. Dietary education in schools, communities, and health facilities in a coherent manner can bring larger effects than stand-alone interventions. Schools can incorporate dietary education and nutritional needs at this age into family-life education or health education.

The school education programme cannot reach out-of-school girls who account for 34% or more in Bangladesh (20). An effective way to reach them and provide nutritional knowledge is by the promotion of behavior-change communication (BCC) between schoolgoing and out-of-school adolescent girls in the community. Community-based BCC may help create an enabling environment for girls in the household and community. BCC may focus on healthy lifestyles as entry-points with information on more sensitive topics, such as human sexuality, sexually transmitted disease, and substance abuse. Periodic assessment of knowledge and practice of girls may guide the discussion issues in accordance with the need. Some operations research may be needed to develop communication materials and about how to sensitize policy-makers, service providers, and teachers to work together and to increase participation in community-based adolescent girls forums.

## ACKNOWLEDGEMENTS

The study used the data from the Baseline Survey 2004 of the National Nutrition Programme (NNP) implemented by a collaborative effort of ICDDR,B, Institute of Public Health Nutrition, and National Institute of Population Research and Training. The NNP, World Bank, and Canadian International Development Agency provided guidance and financial assistance to the baseline survey. The study was funded by ICDDR,B and its donors which provide unrestricted support to the Centre for its operation and research. Current donors providing unrestricted support include: Australian Agency for International Development (AusAID), Government of the People's Republic of Bangladesh, Canadian International Development Agency (CIDA), Embassy of the Kingdom of the Netherlands (EKN), Swedish International Development Cooperative Agency (Sida), Swiss Agency for Development and Cooperation (SDC), and Department for International Development (DFID), UK. The authors gratefully acknowledge these donors for their support and commitment to the Centre's research efforts.

## References

[1] World Bank (2006). Repositioning nutrition as central to development: a strategy for large scale action.

[2] Kurz KM (1996). Adolescent nutritional status in developing countries. Proc Nutr Soc.

[3] Ahmed T, Roy SK, Alam N, Ahmed AMS, Ara G, Bhuiya AU, Shamsul Islam Khan M., Ahmed Tahmeed, Roy Dhaka S.K. (2005). Baseline survey 2004 of the National Nutritional Programme: report.

[4] Ahmed F, Khan MR, Islam M, Kabir I, Fuchs GJ (2000). Anaemia and iron deficiency among adolescent schoolgirls in peri-urban Bangladesh. Eur J Clin Nutr.

[5] Helen Keller International, Bangladesh (1999). Vitamin A status throughout the lifecycle in rural Bangladesh: national vitamin A survey, 1997–98.

[6] Ahmed F, Zareen M, Khan MR, Banu CP, Haq MN, Jackson AA (1998). Dietary pattern, nutrient intake and growth of adolescent school girls in urban Bangladesh. Public Health Nutr.

[7] Abdullah M, Wheeler EF (1985). Seasonal variations, and the intra-household distribution of food in a Bangladeshi village. Am J Clin Nutr.

[8] Ahmad K, Hassan N (1983). Nutrition survey of rural Bangladesh, 1981–82.

[9] Golden MH (1994). Is complete catch-up possible for stunted malnourished children?. Eur J Clin Nutr.

[10] Martorell R, Khan LK, Schroeder DG (1994). Reversibility of stunting: epidemiological findings in children from developing countries. Eur J Clin Nutr.

[11] Public health at a glance—adolescent nutrition 2003.

[12] Kurz KM, Johnson-Welch C (1994). The nutrition and lives of girls in developing countries: findings from the nutrition of adolescent girls research program.

[13] Jamil KM, Rahman AS, Bardhan PK, Khan AI, Chowdhury F, Sarker SA (2008). Micronutrients and anaemia. J Health Popul Nutr.

[14] WHO child growth standards, STATA WHO 2007 package.

[15] de Onis M, Onyango AW, Borghi E, Siyam A, Nishida C, Siekmann J (2007). Development of a WHO growth reference for school-aged children and adolescents. Bull World Health Organ.

[16] Gwatkin DR, Rutstein S, Johnson K, Pande PR, Wagstaff A (2000). Socioeconomic differences in health, nutrition and population.

[17] Shahabuddin AK, Talukder K, Talukder MK, Hassan M, Seal A, Rahman Q (2000). Adolescent nutrition in a rural community in Bangladesh. Indian J Pediatr.

[18] Uddin MJ, Chowdhury MA (2004). Vulnerability to reproductive health of adolescent girls in rural Bangladesh. Fifth Annual Research Conference: proceedings of Population Association of Pakistan, 14–16 December 2004, Karachi.

[19] Levinson J Challenges to improving nutrition adolescent nutrition in Bangladesh and Tanzania.

[20] (2006). Bangladesh educational statistics 2006.

